# Variability in Donor Lung Culture and Relative Humidity Impact the Stability of 2009 Pandemic H1N1 Influenza Virus on Nonporous Surfaces

**DOI:** 10.1128/aem.00633-23

**Published:** 2023-07-05

**Authors:** Zhihong Qian, Dylan H. Morris, Annika Avery, Karen A. Kormuth, Valerie Le Sage, Michael M. Myerburg, James O. Lloyd-Smith, Linsey C. Marr, Seema S. Lakdawala

**Affiliations:** a Tsinghua University School of Medicine, Beijing, China; b Department of Microbiology and Molecular Genetics, University of Pittsburgh School of Medicine, Pittsburgh, Pennsylvania, USA; c Department of Ecology and Evolutionary Biology, Princeton University, Princeton, New Jersey, USA; d Department of Ecology and Evolutionary Biology, University of California, Los Angeles, California, USA; e Center for Vaccine Research, University of Pittsburgh School of Medicine, Pittsburgh, Pennsylvania, USA; f Department of Medicine, Division of Pulmonary, Allergy, and Critical Care Medicine, University of Pittsburgh School of Medicine, Pittsburgh, Pennsylvania, USA; g Department of Civil and Environmental Engineering, Virginia Tech, Blacksburg, Virginia, USA; h Department of Microbiology and Immunology, Emory University, Atlanta, Georgia, USA; Centers for Disease Control and Prevention

**Keywords:** influenza virus, stability, fomite, surface material, relative humidity

## Abstract

Respiratory viruses can be transmitted by multiple modes, including contaminated surfaces, commonly referred to as fomites. Efficient fomite transmission requires that a virus remain infectious on a given surface material over a wide range of environmental conditions, including different relative humidities. Prior work examining the stability of influenza viruses on surfaces has relied upon virus grown in media or eggs, which does not mimic the composition of virus-containing droplets expelled from the human respiratory tract. In this study, we examined the stability of the 2009 pandemic H1N1 (H1N1pdm09) virus on a variety of nonporous surface materials at four different humidities. Importantly, we used virus grown in primary human bronchial epithelial cell (HBE) cultures from different donors to recapitulate the physiological microenvironment of expelled viruses. We observed rapid inactivation of H1N1pdm09 on copper under all experimental conditions. In contrast to copper, viruses were stable on polystyrene plastic, stainless steel, aluminum, and glass, at multiple relative humidities, but greater decay on acrylonitrile butadiene styrene (ABS) plastic was observed at short time points. However, the half-lives of viruses at 23% relative humidity were similar among noncopper surfaces and ranged from 4.5 to 5.9 h. Assessment of H1N1pdm09 longevity on nonporous surfaces revealed that virus persistence was governed more by differences among HBE culture donors than by surface material. Our findings highlight the potential role of an individual’s respiratory fluid on viral persistence and could help explain heterogeneity in transmission dynamics.

**IMPORTANCE** Seasonal epidemics and sporadic pandemics of influenza cause a large public health burden. Although influenza viruses disseminate through the environment in respiratory secretions expelled from infected individuals, they can also be transmitted by contaminated surfaces where virus-laden expulsions can be deposited. Understanding virus stability on surfaces within the indoor environment is critical to assessing influenza transmission risk. We found that influenza virus stability is affected by the host respiratory secretion in which the virus is expelled, the surface material on which the droplet lands, and the ambient relative humidity of the environment. Influenza viruses can remain infectious on many common surfaces for prolonged periods, with half-lives of 4.5 to 5.9 h. These data imply that influenza viruses are persistent in indoor environments in biologically relevant matrices. Decontamination and engineering controls should be used to mitigate influenza virus transmission.

## INTRODUCTION

In 2009, an H1N1 influenza virus emerged from swine and caused a pandemic, with 60.8 million cases in the United States in the first year (1). After its emergence, this 2009 H1N1 pandemic virus (H1N1pdm09) became a seasonal influenza virus, and it continues to circulate globally (2). Seasonal influenza epidemics impose a large public health burden; the Centers for Disease Control and Prevention (CDC) estimated that there were 35 million flu-related illnesses and 20,000 deaths in the United States during the 2019-2020 season (https://www.cdc.gov/flu/about/burden/2019-2020.html). This seasonal burden and the threat of future influenza virus pandemics make it critical to develop strategies to impede influenza virus transmission.

Influenza viruses can spread when infected human hosts expel virus-laden respiratory secretions by breathing, talking, sneezing, or coughing (3, 4). Expelled viruses can contaminate surfaces or objects, and these fomites can infect susceptible individuals—a form of indirect-contact transmission (5–7). Fomites are ubiquitous in household and health care settings. Additionally, the demonstrated environmental stability of infectious influenza viruses in laboratory studies makes fomite transmission of influenza a contributor to the public health burden of influenza viruses (8–13).

Many factors can influence the stability of influenza virus (and therefore its infectivity) on surfaces, including fomite material. Influenza viruses deposited on porous surfaces, such as fabrics and wood, exhibit reduced persistence of infectivity compared to those on nonporous surfaces, including steel and plastic (8, 12, 14, 15). However, influenza virus stability on nonporous surfaces can also depend upon material type; notably, stability on copper is lower than that on stainless steel (16). This observation is consistent with the known antiviral properties of copper (17, 18). More studies are required to determine how influenza virus persists on different materials and could inform engineering controls to mitigate influenza outbreaks.

Environmental conditions such as humidity and temperature also affect the stability of expelled influenza viruses (10, 11, 19). Seasonal fluctuations in relative humidity (RH) have been suggested to be a key factor contributing to seasonal patterns of influenza transmission, in part through the impact of RH on influenza virus environmental stability (20–23). In addition to changes in virus stability, RH can also influence mucociliary clearance and physicochemical properties of expelled secretions (24, 25). Increased humidity can increase the rate at which virus-laden secretions will fall out of the air and be deposited on surfaces. Importantly, respiratory mucus can protect viruses from humidity-mediated decay (26, 27); however, it is still unknown whether virus stability in respiratory mucus on different surface materials is influenced by RH.

The matrix composition of droplets is known to influence viral stability on surfaces. Many prior studies examining influenza virus stability on surfaces used viral stocks propagated in egg allantoic fluid or tissue culture monolayer cell lines, such as Madin-Darby canine kidney cells. The resultant viral suspensions do not mimic the biochemical composition of virus-laden respiratory secretions expelled from an infected individual. Adding exogenous mucin to cell-grown viral stocks has been reported to have no effect on influenza virus stability (10, 11). However, influenza viruses in nasopharyngeal secretions from children with respiratory symptoms were found to be substantially more stable on banknotes than viruses in cell culture medium (28). Similarly, our own recent studies have demonstrated that the presence of respiratory mucus from primary human bronchial epithelial cell (HBE) culture increases the stability of H1N1pdm09 compared to virus suspended in growth media (26, 27). These studies indicate that influenza virus stability needs to be examined further using conditions that more closely mimic respiratory secretions.

While many studies have looked at the individual effects of humidity, surface material, and respiratory droplet composition on influenza virus infectivity, the interplay between these factors remains understudied. A better understanding of this relationship could improve transmission risk assessment and evidence-based mitigation. To address this, here we examined the stability of viruses grown in different HBE cultures on a variety of nonporous materials at RHs ranging from 23% to 98%. The use of HBE cultures better mimics the composition, complexity, and heterogeneity of secretions expelled by infected individuals. We found that virus viability depends not only on the surface tested but also on the RH used. Importantly, we also found that the HBE patient culture influenced viral stability, suggesting that heterogeneity in human respiratory secretions may contribute to persistence of infectious influenza viruses in the environment.

## RESULTS

### H1N1pdm09 stability in droplets is dependent upon surface material.

**FIG 1:**
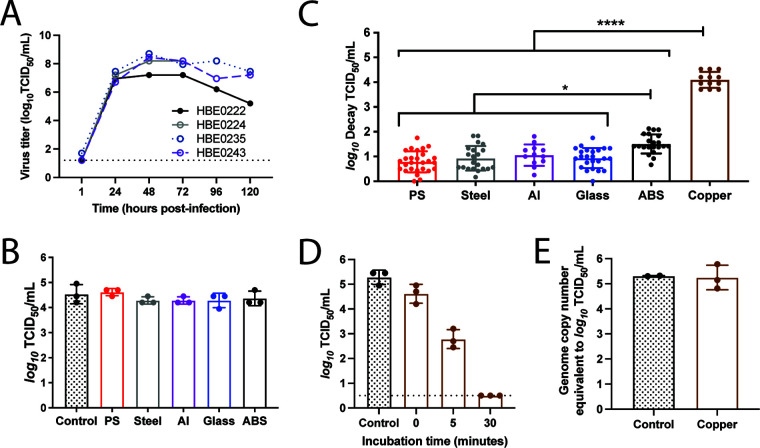
The stability of H1N1pdm09 is dependent on surface material at low relative humidity. (A) Propagation of H1N1pdm09 in four different HBE patient cultures. Each culture can be related to a different pathological state: culture 235 (nonpathological), culture 222 (chronic obstructive pulmonary disease), culture 243 (nonpathological), and culture 224 (idiopathic pulmonary fibrosis). HBE cultures were infected with 10^3^ TCID_50_ of virus per well. Virus was collected at the indicated times and titrated on MDCK cells. Virus samples from 24 to 96 h postinfection for each HBE patient culture were then pooled for use in the stability experiments. (B) Ten 1-μL droplets of H1N1pdm09 were deposited on the indicated surfaces and immediately recovered. Virus titrated as for panel A. Each dot represents individual replicates for each HBE culture. (C) Ten 1-μL droplets of H1N1pdm09 (A/CA/07/2009) were incubated at 23% RH on the surface of the indicated materials for 2 h. Recovered virus was titrated by TCID_50_ assay, and viral decay was expressed as the loss of infectivity compared to a control (10 μL of virus in a sealed Eppendorf tube outside the chamber) that was incubated for the same amount of time. Each data point represents a replicate of H1N1pdm09 propagated from three to four different HBE cultures. Data are means and standard deviations. Asterisks indicate significantly different mean levels of decay between indicated surface materials, as determined by one-way ANOVA with Tukey’s multiple-comparison test (*, *P* < 0.05; ****, *P* < 0.0001). No infectious virus was detected after incubation on copper, and the log_10_ decay values (TCID_50_ per milliliter) were maximal in all replicates. (D) Ten 1-μL droplets of H1N1pdm09 were deposited onto copper. The virus was recovered from the copper surface either immediately after droplet deposition (0 min) or after incubation for 5 or 30 min at room RH, which ranged between 41 and 43%. Virus was titrated by TCID_50_ assay, and data are means and standard deviations. The dashed line indicates the limit of detection. Data shown are representative of three independent experiments in different HBE cultures. (E) Ten 1-μL droplets were recovered following a 2-h incubation on copper at 23% RH. Quantitative PCR for the influenza A virus M gene was used to determine the amount of viral RNA in each sample compared to a known quantity of viral RNA. Each data point in panels C to E represents an individual replicate within a study.

Environmental factors such as temperature, humidity, and the material of the contaminated surface can impact the survival of influenza viruses (29, 30). We propagated H1N1pdm09 in four different HBE patient cultures and collected released virus between 24 and 120 h postinfection (Fig. 1A). HBE patient cultures are derived from primary cells collected from explanted human lung tissue, which are differentiated and maintained at an air-liquid interface (31). Virus collected from 24 to 96 h from each HBE patient culture was pooled and used for subsequent stability analysis. Patient-to-patient variations in the HBE airway surface liquid could influence the stability of viruses; therefore, we performed all studies in all four cultures to look for parameters that influenced virus stability across all cultures.

To assess virus stability, 10 1-μL droplets of HBE-propagated H1N1pdm09 with a starting titer ranging from 10^6.8^ to 10^7^ 50% tissue culture infective doses (TCID_50_)/mL were deposited onto the surface materials of polystyrene (PS) plastic, stainless steel, aluminum, glass, acrylonitrile butadiene styrene (ABS) plastic, and copper. Droplets were then incubated for 2 h inside a desiccator chamber with a RH of 23% to model a dry indoor environment, such as that produced by heating and insulation during a temperate region winter (“flu season,” when influenza spreads efficiently) (23). An equivalent volume of virus stock was incubated in a sealed tube for the same amount of time, to serve as a control. Viable virus was quantified by TCID_50_ assay, and results were compared to those for the control to determine the amount of decay for each sample. Figure 1B demonstrates the similarity between the control samples and droplets deposited onto each surface and immediately recovered. The overall mean of H1N1pdm09 virus decay on each surface was determined using virus propagated in at least three HBE patient cultures (Fig. 1C).

At 23% RH, there was little decay of H1N1pdm09 on PS plastic over the 2-h measurement period (0.79 ± 0.42 log_10_ TCID_50_/mL) relative to the sealed control, as previously observed (26). Similar to results with PS plastic, the mean values of decay on stainless steel, aluminum, and glass were less than or at 1 log_10_ TCID_50_/mL. Decay was significantly higher on ABS plastic (1.5 ± 0.39 log_10_ TCID_50_/mL) and copper, where the deposited virus decayed to undetectable levels (Fig. 1C). The differences in decay did not arise from variable recovery of H1N1pdm09 from the different materials, since immediate recovery of droplets from each surface resulted in titers similar to that of the control (Fig. 1B).

In stark contrast to the moderate decay on ABS plastic after 2 h, no viable H1N1pdm09 was detected after deposition on copper at 23% RH in any replicate, where each replicate reached the maximal decay titer (Fig. 1C). To address whether reduced recovery of H1N1pdm09 from copper impacted the magnitude of decay, droplets were spotted onto the surface and then immediately recovered. Immediate recovery of virus from copper revealed viral titers similar to those of the control sample (Fig. 1D). Additionally, following a 2-h incubation on copper, viral RNA was extracted from recovered virus solution and quantified by qPCR. No difference in total RNA was detected (Fig. 1E), indicating that the rapid inactivation on copper was not due to poor recovery from the material. To determine the longevity of H1N1pdm09 on copper, we shortened the incubation time to 5 or 30 min. Given the short time scale and initial chamber equilibration, H1N1pdm09 droplets were incubated at room RH, which was stable between 41% and 43%. After 5 min on copper, the viability of H1N1pdm09 decreased to 2.78 ± 0.38 log_10_ TCID_50_/mL, whereas a 30-min incubation resulted in a titer that was below the limit of detection (Fig. 1D). Taken together, these data indicate that at 23% RH, H1N1pdm09 is most stable on PS plastic, stainless steel, aluminum, and glass, while it is slightly less stable on ABS plastic and is rapidly inactivated on copper.

### H1N1pdm09 stability is impacted by relative humidity in a surface-dependent manner.

**FIG 2:**
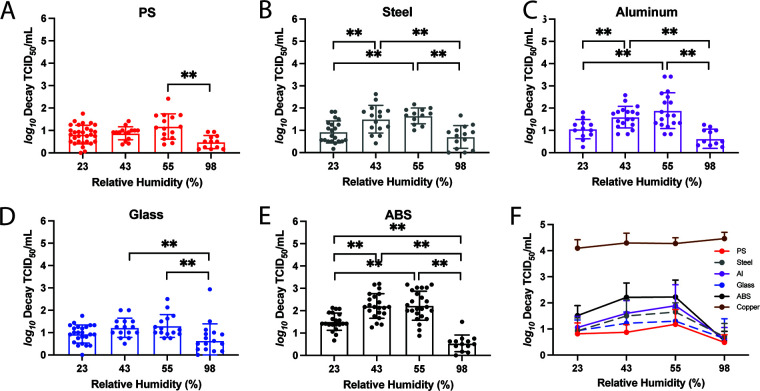
The impact of RH on H1N1pdm09 stability is surface dependent. The stability of H1N1pdm09 in 10 1-μL droplets on each material was tested at a range of RHs: 23%, 43%, 55%, and 98%. The infectivity decay of the virus after 2 h on (A) PS plastic, (B) stainless steel, (C) aluminum, (D) glass, and (E) ABS plastic was calculated as described for Fig. 1. Virus propagated from at least three different HBE cultures was tested under each condition. Each data point represents a single replicate, and the results are means and standard deviations. Two-way ANOVA, using surface material and RH as the variables, with Tukey’s multiple-comparison test was performed on the results in panels A to E to analyze the effect of RH on the virus decay. **, *P* < 0.01 (see also Table 1). (F) The means and standard deviations of virus decay on each of the surfaces in panels A to E and on copper were plotted against the RH.

Prior studies have revealed a relationship between RH and virus stability of enveloped viruses. In many cases, inactivation has been shown to be fastest at midrange RH (about 40 to 70%) compared to low (23%) or high (>80%) RH (19). However, our previously published data demonstrated that H1N1pdm09 is protected from RH-mediated decay in droplets supplemented with airway surface liquid from HBE patient cultures on PS plastic (26). To determine how the stability of HBE-propagated H1N1pdm09 on different surface materials is affected by RH, we spotted 1-μL droplets of H1N1pdm09 on each of the six nonporous surfaces over a range of RH conditions and incubated the droplets for 2 h. In addition to 23% RH shown in Fig. 1, we tested two midrange RHs (43% and 55%), which are typical of indoor environments during summer in temperate regions, and one high RH (98%), which mimics conditions during rainy periods and in airways. Viable H1N1pdm09 was recovered from PS plastic, stainless steel, aluminum, glass, and ABS plastic at all RHs tested, but the magnitude of decay varied by both RH and surface material (Fig. 2A to E). Overall, little decay was observed on PS plastic and glass at 23%, 43%, and 55% RH, with viruses being more stable at 98% RH than 43% and 55% RH (Fig. 2A and D). H1N1pdm09 displayed maximal decay on stainless steel and aluminum at the midrange RHs but was more stable at both 23% and 98% RH (Fig. 2B and C). Decay of H1N1pdm09 on ABS plastic was the highest at midrange humidities, followed by 23% RH, which showed significantly more decay than 98% RH (Fig. 2E; Table 1). Similar to what was seen at 23% RH, no infectious virus was detected on copper surfaces after 2 h under all other RH conditions (Fig. 2F).

**TABLE 1:** Statistical analysis of the surface-based variations in the stability of H1N1pdm09 in droplets at different RH from Fig. 2

RH (%)	Surface material	Statistical significance (*P*) of differences in virus decay on surface^*a*^			
		PS plastic	Glass	Steel	Aluminum
23	ABS plastic	<0.0001	0.0023	0.0027	NS
	Aluminum	NS	NS	NS	
	Steel	NS	NS		
	Glass	NS			
43	ABS plastic	<0.0001	<0.0001	0.0005	0.0021
	Aluminum	0.0007	NS	NS	
	Steel	0.0089	NS		
	Glass	NS			
55	ABS plastic	<0.0001	<0.0001	0.0146	NS
	Aluminum	0.0010	0.0098	NS	
	Steel	NS	NS		
	Glass	NS			
98	ABS plastic	NS	NS	NS	NS
	Aluminum	NS	NS	NS	
	Steel	NS	NS		
	Glass	NS			

a Adjusted *P* values after Tukey’s multiple-comparison test are listed. Refer to Fig. 2 for corresponding data sets of mean virus decay observed from multiple HBE culture replicates under the indicated conditions. NS, not significant (*P* > 0.05).

Direct comparison of virus decay by surface reveals a hierarchy of viral persistence by surface type (Fig. 2F). Viruses appear most stable on PS plastic and glass surfaces, with increased decay on stainless steel and aluminum, followed by ABS plastic. No surface-based differences in virus decay were observed at 98% RH where the mean decay values were all below 1 log_10_ TCID_50_/mL, even on ABS plastic (Fig. 2F; Table 1). Taken together, our results demonstrate that RH can affect the stability of HBE-propagated H1N1pdm09, but this feature was apparent only on certain surface materials. Additionally, certain nonporous materials exhibit more viral destabilization than others.

### Predicted half-life of virus depends more strongly on HBE culture than on surface material.

Our experiments used virus propagated from different HBE cultures derived from individual patients (Fig. 1A). Patient-to-patient variability in the HBE airway surface liquid surrounding the released virions might also contribute to differences in viral stability on various materials. Therefore, we wanted to examine the relationship between surface material and HBE composition on virus stability. To do this, we spotted HBE-propagated H1N1pdm09 droplets (from the four distinct patient cultures) for 2, 8, or 24 h on PS plastic, stainless steel, glass, and ABS plastic. To mimic a dry indoor environment in winter, H1N1pdm09 droplets were incubated in a chamber at 23% RH. The recorded RH inside the chamber was stable for up to 24 h after a brief equilibration period during the first few minutes. Viral titers were assessed after recovery of the droplets at each time interval and then used to calculate the half-life of each HBE-grown virus.

**FIG 3:**
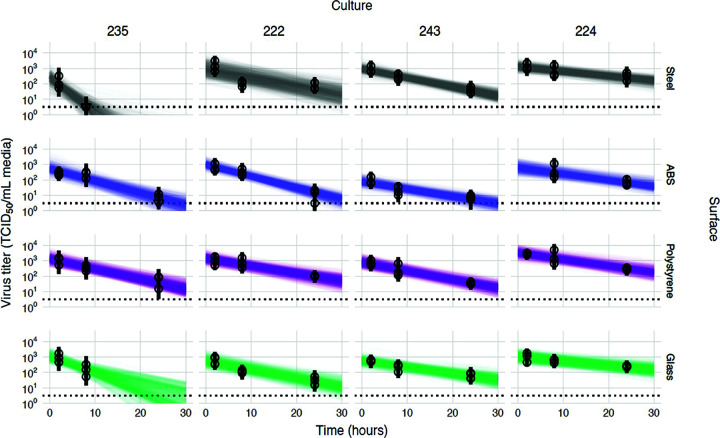
Linear regression of viral stability on surfaces at 23% RH over time by HBE culture to estimate surface and culture effects. Regression lines representing predicted exponential decay of log_10_ virus titer over time are plotted alongside measured (directly inferred) virus titers. Predicted decay reflects the estimated effects of surface (row) and culture (column). For each experiment (surface/HBE culture pair), semitransparent regression lines visualize the inferred joint posterior distribution of the virus exponential decay rate and the individual sample intercepts (i.e., virus titers at time zero, which can vary about the mean initial titer for the experiment). Fifty random posterior draws are shown. To visualize the estimated variation in initial titers (intercepts), 6 lines are plotted per experiment per draw, one for each of 6 randomly chosen titers from that experiment (since each titer has its own estimated time zero value). This yields 300 plotted lines per experiment. A new set of 6 random titers per experiment was chosen for each draw. Points with black bars show individually estimated titers (point, posterior median titer estimate; bar, 95% credible interval). Samples with no positive titration wells are plotted as triangles at the approximate limit of detection (dotted horizontal line).

**FIG 4:**
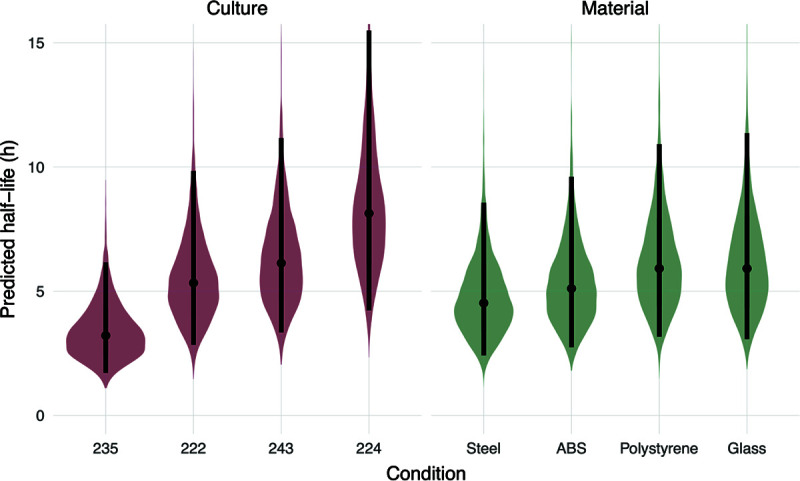
Predicted half-lives based on surface material or patient-derived culture. Violin plots indicate the posterior distributions of the half-life of viable virus based on the estimated exponential decay rates of the virus in different HBE cultures on a neutral surface (left) and on surfaces with a neutral HBE culture (right), thus separating culture and surface effects. Dots indicate the posterior median estimates, and the black lines indicate a 95% credible interval (CI). Values for the median half-lives and 95% CI are presented in Table 2.

To estimate the half-life of viable influenza virus and its dependence on surface and HBE culture, we used a hierarchical regression model adapted from our own previously published Bayesian statistical methods for analyzing viral environmental stability (32, 33). Briefly, we inferred virus half-lives and surface- and culture-level effects upon those half-lives directly from raw titration data (inoculated wells positive or negative for virus infection). Initially, we analyzed all the data together, using a model that assumes that surface and culture act independently (and multiplicatively) to modify the half-life. The models quantify virus from positive or negative readouts of inoculated wells by treating well inoculation as a “single-hit” process, with an assumed Poisson distribution for the number of virions. This is similar to traditional endpoint titration statistical approaches such as the Spearman-Karber and Reed-Muench methods. However, implementing this in a Bayesian framework connects our model directly to a regression for estimating half-lives, and we can obtain principled estimates of uncertainty (such as 95% credible intervals) for each individual titration, rather than just a single number. The resulting model fits for each culture and surface are displayed in Fig. 3, and the resultant estimates of culture and surface effects are shown in Fig. 4.

To estimate virus half-lives, we coupled our titer estimation model to a simple regression model in which virus is assumed to decay exponentially over time. The slope of individual regression lines predicting titer as a function of time will provide an estimate of the exponential decay rate, which can be converted directly into a half-life. To estimate the effects of individual surfaces and cultures on virus persistence, we used a regression model in which we treated log half-life of the virus in a given culture on a given surface as depending on three quantities: a culture effect (assumed to apply across surfaces), a surface effect (assumed to apply across cultures), and a single intercept (representing the average log half-life across all cultures and surfaces tested). This simple model assumes that the culture effect and surface effect act independently to modify the half-life of the virus.

Using this approach on our experimental data, we report the predicted half-life for the virus in each given culture on a hypothetical “neutral” (average) surface—that is, with zero surface effect. Similarly, we report estimated surface effects as predicted half-lives on each surface given a hypothetical neutral (average) culture—that is, with zero culture effect (Fig. 4 and Table 2).

**TABLE 2:** Virus half-life by surface material and HBE culture

Criterion	Median virus half-life (95% credible interval)
Surface material	
Stainless steel	4.52 (2.41, 8.56)
ABS plastic	5.10 (2.74, 9.60)
PS plastic	5.91 (3.17, 10.9)
Glass	5.91 (3.07, 11.4)
HBE culture	
Culture 235	3.21 (1.71, 6.16)
Culture 222	5.33 (2.84, 9.84)
Culture 243	6.13 (3.34, 11.2)
Culture 224	8.13 (4.23, 15.5)

The median viral half-life of a neutral culture on different surface materials (excluding copper) ranged between 4.5 and 5.9 h (Table 2). In contrast, the median viral half-life range on a neutral surface broken down by HBE culture was 3.2 to 8.1 h (Table 2). The greater estimated variation in half-lives by HBE culture than by surface material suggests that host-specific variation may be an important determinant of environmental transmission risk. Calculations of virus half-life for each HBE culture include data from all surface materials (excluding copper). However, given the similarity of virus half-life values across surface material type, it is likely that the large variations observed between virus half-life by HBE culture are not due to surface material but rather differences in the airway secretion composition.

## DISCUSSION

In this study, we investigated influenza virus stability on different types of surfaces using H1N1pdm09 propagated from three-dimensional patient-derived lung cultures. Stability of H1N1pdm09 in the presence of airway surface liquid appears to be surface dependent under various common indoor RHs tested (23%, 43%, and 55%). The notable exception was deposition on copper, which completely inactivated virus at all RH levels and as quickly as 30 min at room RH. On the other nonporous surfaces tested (excluding copper), the half-life of HBE-propagated H1N1pdm09 at 23% RH was similar on PS plastic, stainless steel, aluminum, and glass. Interestingly, a subsequent analysis revealed that the half-life of infectious H1N1pdm09 at 23% RH varied substantially among the different HBE patient cultures that were used to propagate the virus. This final observation suggests that the respiratory secretion composition may have a profound impact on the persistence of viruses in the environment and that individual variation may matter a great deal.

The rapid inactivation of HBE-propagated H1N1pdm09 observed on copper at all RHs tested indicates that this effect is mediated by the surface itself and is robust against other environmental conditions. Noyce et al. also described inactivation of 20-μL droplets of A/PR/8/34 (H1N1) after 6 h on a copper surface (16), whereas in our study, 1-μL droplets of H1N1pdm09 had no detectable infectious virus after just 30 min. The size and composition of the influenza virus-containing droplets deposited on a given surface may impact virus viability and decay kinetics. Virucidal activity of copper and copper alloys has been reported for seasonal coronaviruses, severe acute respiratory syndrome coronavirus 2 (SARS-CoV-2), and norovirus (17, 34–37). The potent antiviral properties of copper were shown by Warnes et al. to damage membrane and surface proteins and to produce nonspecific fragmentation of the coronavirus 229E genome (18). Previous studies have also shown that bacteria are rapidly killed on dry copper surfaces (38), which has been proposed to be mediated by DNA damage (39, 40). Addition of copper oxide or copper nanocompounds to frequently touched surfaces could reduce the spread of influenza (41–43).

Environmental conditions such as RH could affect the transmissibility of influenza virus fomites by influencing virus stability. Relevant RH conditions include those similar to indoor RH during temperate-zone winters (approximately 20%) and those similar to indoor RH during temperate-zone summers (40 to 60%). Previous work has suggested a U-shaped relationship between RH and virus stability for enveloped viruses, including SARS-CoV-2 and influenza virus, where virus stability is lowest at midrange RHs (19, 33). However, our studies with influenza viruses suspended in HBE airway surface liquid have revealed that viruses are protected from RH-mediated decay on PS plastic (26). In this study, we reproduced our previous observations that HBE-propagated H1N1pdm09 was stable on PS plastic (Fig. 2). However, we also observed variable stability of H1N1pdm09 propagated in HBE patient cultures at 23%, 43%, and 55% RH on stainless steel, aluminum, and ABS plastic nonporous surfaces (Fig. 2). These data suggest that RH-mediated decay of influenza virus may be more pronounced on different surfaces and may also depend on the composition of the respiratory droplet.

Most surprisingly, the half-lives of HBE-propagated influenza viruses at 23% RH appeared similar across surfaces, but droplet composition from HBE cultures impacted H1N1pdm09 stability more (Fig. 4). This suggests a key role for droplet composition in the persistence of viruses in the environment, although the mechanisms by which virus stability is altered are unclear. Analyzing potential changes in viral structures between HBE cultures could prove useful in identifying culture composition features that affect virus infectivity. Our cultures included those from normal (nonpathological) lung transplants (cultures 235 and 243) and those with disease states (cultures 222 and 224). More experiments utilizing virus grown from greater numbers of HBE cultures will also be required to determine if and how pathological states contribute to virus stability. Additionally, further studies examining the differences in composition and volume of expelled droplets across individuals could yield insight into the observed heterogeneity of influenza transmission (44). The SARS-CoV-2 pandemic was driven by cluster-based transmission: 20% of infected individuals seeded 80% of secondary infections (45). This suggests that individual characteristics of “superspreaders” may impact the emergence of novel respiratory viruses (46, 47). Understanding which respiratory secretion components are beneficial or harmful to virus stability, and how these map to identifiable traits of human individuals, could aid in understanding what makes a superspreader.

The emergence of multiple respiratory pandemic viruses in the last few decades has highlighted the importance of understanding virus transmission pathways (inhalation of aerosols, spray of large droplets, and touching of fomites) and of determining the impact of virus traits, environmental conditions, and host factors on each mode (48). Persistence of infectious influenza virus on various nonporous surfaces over extended time periods indicates that regular decontamination of frequently touched surfaces, in combination with engineering controls such as indoor RH control, could be effective nonpharmaceutical interventions to limit fomite transmission of influenza viruses. Patterns of heterogeneous virus stability by individual patient culture suggest a potential contributing mechanism to observed host heterogeneity in virus transmission.

## MATERIALS AND METHODS

### Cells and virus.

Madin-Darby canine kidney (MDCK) cells (ATCC) were maintained in Eagle’s minimum essential medium supplemented with 10% fetal bovine serum, 2 mM l-glutamine, and 1% penicillin/streptomycin. Four primary human bronchial epithelial cell (HBE) cultures were differentiated from lung tissues of four different patients and were maintained at an air-liquid interface in Transwells (31). Each HBE culture was derived from patient lungs with various pathological states: culture 235 (nonpathological), culture 222 (chronic obstructive pulmonary disease), culture 243 (nonpathological), and culture 224 (idiopathic pulmonary fibrosis).

The 2009 pandemic H1N1 influenza A virus, A/California/07/2009 (H1N1pdm09), was acquired as previously described (26). To prepare HBE-propagated virus stock, 3 log_10_ TCID_50_ of virus was added to each Transwell from the apical side after the cells had been washed once with phosphate-buffered saline (PBS). The inoculum was removed after 1 h, and the HBE-propagated virus was collected with 150 mL of PBS every 24 h up to five times. Viral washes collected at the same time from different Transwells of the same HBE culture were pooled and titrated on MDCK cells. Up to four viral washes with the highest titers from the same HBE culture were combined. To make an abundant HBE-propagated virus stock without diluting physiological components in the suspension, the combined virus wash was diluted 1:1 with the cell wash from the same HBE culture, which had been collected prior to infection. To account for patient-specific variations, four stocks of HBE-propagated virus from different cultures were made. The stocks were aliquoted and stored at −80°C before use.

### Surface preparation.

Six surface materials were selected because of common use: PS plastic, stainless steel, aluminum, glass, ABS plastic, and copper. Disposable PS plastic was readily available as the flat bottom of a 6-well tissue culture plate (TPP; Sigma). Disposable glass cover slides (22CIR-1; Thermo Fisher) were used as glass surfaces without additional preparation. Circular, smooth-surface coupons of 304 stainless steel (20 gauge), 6061 aluminum (1.6 mm thick), ABS plastic (black, 0.6 mm thick), and 110 copper (0.6 mm thick) with a uniform diameter of 20 mm were purchased through Alumagraphics. The coupons were cleaned with an interfering-residual-free detergent (Alconox) prior to sterilization (stainless steel, aluminum, and glass) or by disinfection with isopropanol (ABS plastic). The coupons were reused after cleaning and sterilization (or disinfection) following the assay.

### Virus stability assay.

Saturated saline solutions were placed in a sealed chamber to condition the interior relative humidity (RH) to desired levels: KCH_3_COO for 23%, K_2_CO_3_ for 43%, Mg(NO_3_)_2_ for 55%, and K_2_SO_4_ for 98% (19). The temperature and RH inside the chamber were monitored by a HOBO logger. Ten 1-μL droplets of the HBE-propagated virus were pipetted either directly onto each well (PS plastic) or onto a coupon inside a lidless 6-well plate in triplicate. The plate was immediately transferred into the RH chamber upon droplet deposition. After incubation for 0.5, 2, 8, or 24 h, the virus was collected by rinsing each well, or each coupon, with 1 mL Leibovitz’s L-15 medium, resulting in a 1:100 dilution compared with the volume of the deposited droplets. The control sample was a bulk 10-μL suspension of the same HBE-propagated virus in a 1.5-mL Eppendorf tube, which was incubated for the same amount of time without RH chamber conditioning or contact with the coupons. The RH chamber was not used for shorter virus incubation on copper coupons, since the chamber required at least 5 min to equilibrate the interior RH. Instead, the virus was incubated on copper coupons inside a lidless 6-well plate at room RH. Either immediately (0 min) or after 5 or 30 min of incubation, the virus was recovered as described above. The control sample was immediately diluted without incubation. All procedures were conducted inside a biosafety cabinet at room temperature (22 to 24°C). Under each condition, HBE-propagated H1N1pdm09 from at least three different cultures was tested.

The decay of H1N1pdm09 estimated in Fig. 1 and 2 is defined as loss of infectivity as determined by TCID_50_ assay (49). Specifically, the virus samples were titrated by 10-fold serial dilutions on a 96-well plate of confluent MDCK cells in quadruplicate. A 24-well plate was also used where undiluted virus samples were incubated on MDCK cells in duplicate to provide the limit of detection at 0.5 log_10_ TCID_50_/mL. The loss of virus infectivity was then calculated by comparing titers of the virus on the surface coupons with titers of the control samples incubated for the same amount of time: log_10_ decay TCID_50_ = log_10_ (TCID_50_ of the control/TCID_50_ of virus on surfaces).

The results of the virus decay were analyzed with GraphPad Prism 8. Statistical analysis excluded results from copper, because the virus decay was mostly beyond the detection limit. At 23% RH, one-way analysis of variance (ANOVA) using surface material as the variable was performed with Tukey’s multiple-comparison test to define surface-based variations in virus decay within 2 h. Two-way ANOVA using RH (or incubation time) as the second variable was performed with Tukey’s multiple-comparison test to define the effect of RH (or incubation time) on virus decay. Due to the interactive effects of RH and surface material on virus decay, the analysis of surface-based variations at different RH was adjusted with Tukey’s multiple-comparison tests following two-way ANOVA and summarized in Table 1.

### qRT-PCR.

The RNA of H1N1pdm09 was extracted from virus samples using a PureLink viral RNA/DNA minikit (Invitrogen). For quantitative reverse transcription-PCR (qRT-PCR), 5 μL of the viral RNA was analyzed using a TaqMan RNA-to-*C_T_* 1-step kit (Thermo Fisher). Primers specific to the matrix gene (M) were used at a concentration of 0.1 μM (M25 F [5′-AGA TGA GTC TTC TAA CCG AGG TCG-3′] and M123 R [5′-GC AAA GAC ATC TTC AAG TCT CTG-3′]) and an M64 probe (6-carboxyfluorescein [FAM]-TCA GGC CCC CTC AAA GCC GA-NFQ) was used at 0.25 μM. All reactions were performed in duplicate. The thermal cycling step was conducted on a StepOnePlus real-time PCR system (Applied Biosystems) with StepOne software (version 2.3) following the manufacturer’s recommendations. A standard curve was generated with five 10-fold serial dilutions of viral RNA extracted from a stock virus with a known TCID_50_ titer.

### Bayesian inference methods.

To estimate the effects of surface and culture on virus half-lives (Fig. 3 and 4), we jointly inferred these effects and the corresponding half-lives directly from raw titration data (inoculated wells positive or negative for virus infection), using Bayesian inference with a suite of custom statistical models. Below, we provide a conceptual overview of those models, followed by a full technical description. We have also published code implementing the model for reproducibility.

The models quantify virus from positive or negative readouts of inoculated wells by treating well inoculation as a “single-hit” process: if at least one virion successfully infects a cell within the well, the well will show evidence of infection. We then estimate the virus concentration in TCID_50_ from the distribution of positive and negative wells observed at various dilutions of the sample. We assume that the number of virions inoculated into any given well is Poisson distributed, with a mean given by the virus concentration in the diluted sample used for that well. This is the same Poisson single-hit assumption that underlies traditional endpoint titration statistical approaches, such as the Spearman-Karber and Reed-Muench methods. The difference is that by implementing this in a Bayesian framework, we are able to connect our model directly to a regression for estimating half-lives, and we are able to obtain principled estimates of uncertainty (such as 95% credible intervals) for each estimated quantity, rather than just a single number.

To estimate virus half-lives, we coupled our titer estimation model to a simple regression model in which virus is assumed to decay exponentially over time. The slope of a regression line predicting log titer as a function of time therefore gives an estimate of the exponential decay rate, which can be converted directly into a half-life. Our Bayesian approach allows us to account in a principled way for various sources of noise, such as variation in the initial quantities of virus deposited onto individual surface coupons.

Finally, to estimate the effects of individual surfaces and cultures on virus persistence, we used a regression model in which we treated log half-life of the virus in a given culture on a given surface as depending on three quantities: a culture effect (assumed to apply across surfaces), a surface effect (assumed to apply across cultures), and a single intercept (representing the average log half-life across all cultures and surfaces tested). The model assumes that the culture effect and surface effect act independently to modify the half-life of the virus. We estimated these culture and surface effects, and the intercept, from our data.

To aid interpretability, we report estimated culture effects in units of half-life. Specifically, we report the predicted half-life for the virus in the given culture on a neutral (average) surface—that is, with zero surface effect. Similarly, we report estimated surface effects as predicted half-lives on the given surface given a neutral (average) culture—that is, with zero culture effect.

**(i) Notation.** In the model notation that follows, the symbol “~” indicates that a random variable is distributed according to the given distribution. Normal distributions are parameterized as ~normal (mean, standard deviation). Positive-constrained normal distributions (Pos-normal) are parameterized as ~Pos-normal (mode, standard deviation). Beta distributions are parameterized as ~beta (α, β), with canonical shape parameters α and β > 0 (which can be thought of as 1 plus the number of prior successes and failures, respectively, in a set of α + β binomial trials).

**(ii) Titer inference and model description.** We inferred individual titers directly from titration well data according to a Poisson single-hit model (32), as described in reference 33.

Briefly, we assume that individual wells for sample *i* are positive if at least one virion successfully infects a cell. We assume the number of virions that successfully infect cells within a given well is Poisson distributed with a mean given by the concentration of viable virions in the plated sample.

This gives us our likelihood function, assuming independence of outcomes across wells. Titrated doses introduced to each cell-culture well had a volume of 0.1 mL, so we incremented inferred titers by 1 to convert to units of log_10_ TCID_50_ per milliliter.

The one variation from the model described in reference 33 is that here we also had negative-control wells. We used these to estimate the probability of a false-positive well. Whereas in reference 33 the mean of our Poisson was given by $\ln(2)10^{v_{i}}$, where *v_i_* is the concentration of viable virus in TCID_50_ per 0.1 mL, here we have a mean of $\ln(2)10^{v_{i}}+\;f$, where *f* is a constant governing the false-positive rate and is related to the probability of a false positive as follows: *p_fp_* = 1 − exp(−*f*). Therefore, *f* = −ln(1 − *p_fp_*).

**(iii) Prior distributions.** We assigned a weakly informative normal prior to the log_10_ titers *v_i_* (the titer for sample *i* measured in log_10_ TCID_50_ per 0.1 mL, since wells were inoculated with 0.1 mL): *v_i_* ~ normal(2.5, 3). We placed a beta prior on the false-positive probability *p_fp_*, assuming it to be small but allowing it to be nontrivial: *p_fp_* ~ beta (1, 50).

**(iv) Predictive checks.** We assessed the appropriateness of prior distribution choices using prior predictive checks. The prior checks suggested that prior distributions were agnostic over the titers of interest.

### Half-life inference model.

**(i) Model description.** To infer half-lives of viable virus in the various experiments, we used a regression model.

For each experimental condition *i*, we have two sets of measurements: those for treatment samples deposited on surfaces and incubated at a given temperature and humidity, and those for control samples kept in bulk solution at room temperature. We assume that each sample *j* for experimental condition *i*, whether treatment or control, had some unknown initial log_10_ titer *v_ij_*_0_ at time zero.

We assume that all of these initial values are normally distributed about a mean initial log_10_ titer ${\bar{v}}_{i0}$ for the experiment, with an unknown experiment-specific standard deviation σ*_vi_*: *v_ij_*_0_ ~ normal (${\bar{v}}_{i0}$, σ*_vi_*).

We modeled loss of viable virus as exponential decay at a rate δ*_i_* for treatment samples in condition *i* and λ*_i_* for the corresponding control samples. For the treatment samples, we assumed there was an experiment-specific mean amount of virus lost during deposition (ℓ*_i_*).

It follows that the quantity *v_ij_* of virus sampled at time *t_ij_* is given by

$$v_{\mathrm{ij}}=\{\begin{array}{cc} v_{\mathrm{ij}0}\;-\;\ell_{i}\;-\;\delta_{i}t_{\mathrm{ij}} & \;\mathrm{ij}\text{ is a treatment sample}\\ v_{\mathrm{ij}0}\;-\;\lambda_{i}t_{\mathrm{ij}} & \mathrm{ij}\text{ is a control sample} \end{array}$$

We then used the direct-from-well data likelihood function described above, except that instead of estimating individual titers independently, we estimated the values of δ*_i_*, λ*_i_*, and ℓ*_i_* under the assumption that our observed well data reflected the corresponding predicted titers *v_ij_*.

**(ii) Prior distributions.** We placed a weakly informative normal prior on the mean initial log_10_ titers ${\bar{v}}_{i0}$ to reflect the known inocula: ${\bar{v}}_{i0}$ ~ normal (3.5, 1). We placed a Pos-normal prior on the initial titer standard deviations σ*_vi_*: σ*_vi_* ~ Pos-normal (0, 0.2). This allows either for large variation (more than ±0.5 log_10_) about the experiment mean or for substantially less variation, depending on the data.

We placed normal priors on the log treatment and control half-lives Δ*_i_* and Λ*_i_*, where Δ*_i_* = ln{[log_10_(2)]/δ*_i_*} and Λ*_i_* = ln{[log_10_(2)]/λ*_i_*}. We made the priors weakly informative (diffuse over the biologically plausible half-lives); we verified this with prior predictive checks: Δ*_i_* ~ normal [ln (5), 2] and Λ*_i_* ~ normal [ln (24), 3].

We placed a Pos-normal prior on the experiment mean deposition losses (ℓ*_i_*): ℓ*_i_* ~ Pos-normal (0.5, 0.5).

The prior for the false-positive probability *p_fp_* was as in the titer estimation model (see “Titer inference and model description”).

**(iii) Predictive checks.** We assessed the appropriateness of prior distribution choices using prior predictive checks and assessed goodness of fit for the estimated model using posterior predictive checks.

### Model to estimate culture and surface effects. (i) Model description.

To estimate and compare the effects of surface and culture on virus persistence, we used a simple regression model. We estimated treatment and control log half-lives Δ*_i_* and Λ*_i_* as in the half-life inference model (detailed above), but instead of placing priors directly on them, we assumed that the log half-lives Δ*_i_* could be predicted according to a linear equation with intercept $\bar{\Delta }$, culture-specific effects χ*_n_*, and surface-specific effects μ*_m_*: Δ*_i_* = $\bar{\Delta }$ + χ*_C__(i)_* + μ*_S__(i)_* + ϵ*_i_*, where $\bar{\Delta }$ is an intercept representing the log half-life for virus in a neutral (zero-effect) culture on a neutral (zero-effect) surface, *C*(*i*) is the culture for experiment *i*, *S*(*i*) is the surface for experiment *i*, and ϵ*_i_* is a normally distributed error term with an estimated standard deviation σ_Δ_: ϵ*_i_* ~ normal (0, σ_Δ_).

We assumed that the control log half-lives Λ*_i_* were normally distributed about an unknown mean $\bar{\Lambda }$ with an unknown standard deviation σ_Λ_: Λ*_i_* ~ normal ($\bar{\Lambda }$, σ_Λ_).

### (ii) Prior distributions.

We placed normal priors on the intercept of the log half-life for a neutral surface, $\bar{\Delta }$, and the mean control log half-life, $\bar{\Lambda }$, equivalent to the priors used previously for Δ*_i_* and Λ*_i_* in the half-life inference model: Δ*_i_* ~ normal [ln (5), 2] and Λ*_i_* ~ normal [ln (24), 3].

We used normal priors centered on zero for the culture effects χ*_n_* and the surface effects μ*_n_* with standard deviations designed to rule out implausibly large effects: χ*_n_* ~ normal (0, 0.5) and μ*_n_* ~ normal(0. 0.5).

We placed Pos-normal priors on the standard deviations σ_Δ_ and σ_Λ_: σ_Δ_ ~ Pos-normal(0, 0.33) and σ_Λ_ ~ Pos-normal (0, 0.33).

These allow either for substantial (1-log) variation compared to the regression prediction or for minimal variation.

All other priors were as in the half-life estimation model.

### (iii) Predictive checks.

We assessed the appropriateness of prior distribution choices using prior predictive checks and assessed goodness of fit for the estimated model using posterior predictive checks.
